# Lignin Derived Free‐Standing Sulfur Host Functionalized with MoS_2_ for Li–S Batteries

**DOI:** 10.1002/cssc.202501698

**Published:** 2025-10-03

**Authors:** Ping Feng, Qingping Wu, Yael Rodriguez Ayllon, Yongchao Chen, Marius Hermesdorf, Martin Oschatz, Yan Lu

**Affiliations:** ^1^ Institute of Electrochemical Energy Storage Helmholtz‐Zentrum Berlin für Materialien und Energie 14109 Berlin Germany; ^2^ Institute for Technical Chemistry and Environmental Chemistry Friedrich‐Schiller‐Universität Jena 07743 Jena Germany; ^3^ Chongqing Institute of Green and Intelligent Technology Chinese Academy of Sciences Chongqing 400714 China; ^4^ Helmholtz Institute for Polymers in Energy Applications Jena (HIPOLE Jena) Lessingstraße 12‐14 07743 Jena Germany

**Keywords:** electrospinning, free‐standing electrodes, lignin, lithium−sulfur batteries, polysulfide conversion

## Abstract

Lignin, a widely available natural polymer and sustainable biomass precursor, contains over 60 wt% carbon. However, its potential for producing high‐value carbon‐based materials and its application in energy‐related areas remain largely underutilized. In this work, a green and scalable strategy is reported for constructing a free‐standing carbon nanofiber (CNF) film by electrospinning using lignin as the carbon precursor, with uniformly embedded MoS_2_ nanoparticles to endow integrated catalytic function. The resulting MoS_2_/CNFs film exhibits excellent structural integrity, enabling its direct use as a binder‐free and current‐collector‐free cathode framework for lithium–sulfur (Li−S) batteries. Furthermore, the embedded catalytic components can chemically adsorb lithium polysulfides and enhance sulfur redox reaction kinetics. As a result, Li−S cells with MoS_2_/CNFs‐based cathode demonstrate excellent cycling stability, maintaining a capacity of 609.3 mAh g^−1^ after 200 cycles at 1C. This work highlights a promising approach for transforming low‐cost lignin into multifunctional electrode materials, offering both structural robustness and catalytic activity for next‐generation Li–S batteries.

## Graphical abstract

Lignin, a sustainable carbon‐rich biomass, remains underutilized for energy applications. Here, a green electrospinning strategy is developed to fabricate free‐standing carbon nanofiber (CNF) films from lignin with uniformly embedded MoS_2_ nanoparticles. The resulting MoS_2_/CNFs serve as binder‐free cathodes for Li−S batteries, offering strong polysulfide adsorption, fast redox kinetics, and stable cycling (609.3 mAh g^−1^ after 200 cycles at 1C).

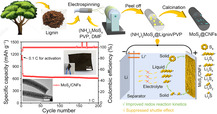

## 1 Introduction

The growing emphasis on sustainable development and environmental protection has generated significant interest in the high‐value utilization of lignocellulosic biomass as an alternative to nonrenewable petroleum‐based chemicals.^[^ ^1^ ^]^ Lignocellulosic biomass primarily consists of three major organic components: cellulose, hemicellulose, and lignin.^[^ ^2^ ^]^ Cellulose is extensively used in the papermaking and biorefinery industries, while hemicellulose can be converted into valuable chemicals such as lactic acid and furfural.^[^ ^3^ ^]^ In contrast, lignin is considered a low‐value by‐product in these industries.^[^ ^4^ ^]^ As the second most abundant natural macromolecule on earth, lignin has an estimated annual commercial availability of up to 70 million tons.^[^ ^5^ ^]^ However, ≈95% of this lignin is directly burned for energy generation.^[^ ^6^ ^]^ There is an increasing demand for establishing commercially relevant use cases for this material. The conversion of lignin to functional carbon materials would be one possible way to chemically upgrade it into compounds that are applicable, for instance, in future energy storage concepts.

Carbon materials are widely employed in energy and environmental applications due to their excellent electrical conductivity in combination with chemical and mechanical stability, large specific surface area, and tunable pore structures.^[^ ^7^ ^]^ Currently, commercial carbon materials are primarily derived from petroleum refining byproducts such as petroleum coke and pitch, as well as petroleum‐based polymers like polyacrylonitrile.^[^ ^8^ ^]^ Given its high carbon content (>60 wt%), low cost, and renewable nature, lignin represents a promising precursor for the synthesis of carbon‐based materials.^[^ ^9^ ^]^ Through high‐temperature pyrolysis, lignin can be converted into carbon nanofibers (CNFs), carbon nanoparticles, and heteroatom‐doped carbon.^[^ ^10^ ^]^ Utilizing lignin as a low‐cost precursor to produce various carbon materials presents an opportunity to transform it from an industrial byproduct into high‐value‐added materials while simultaneously reducing the production costs of carbon‐based materials.^[^ ^11^ ^]^

Since Nazar et al. first introduced ordered mesoporous carbon (CMK‐3) as a sulfur host in 2009, demonstrating excellent cycling stability through the physical confinement of sulfur, various mesoporous and microporous carbon structures have been investigated for Li–S batteries.^[^ ^12^ ^]^ Lithium–sulfur (Li–S) batteries have recently garnered significant attention as next‐generation energy storage systems due to their exceptionally high theoretical energy density (up to 2600 Wh kg^−1^), high theoretical specific capacity (1675 mAh g^−1^), and predicted cost‐effectiveness.^[^ ^13^ ^]^ However, the commercialization of Li–S batteries faces several critical challenges, including (a) the intrinsically poor electrical conductivity of sulfur and significant volume expansion (≈80% during full lithiation to Li_2_S); (b) severe capacity degradation due to the dissolution of intermediate lithium polysulfides (LiPSs) during cycling; and (c) sluggish kinetics of solid–liquid–solid conversion reactions.^[^ ^14^ ^]^ To address these issues, researchers have explored various strategies for the design of advanced sulfur host materials; including 1) embedding sulfur into porous carbon frameworks to enhance conductivity and structural stability of sulfur cathodes;^[14a]^ 2) incorporating polar sulfur hosts, such as heteroatom‐doped carbon, metal oxides, and metal sulfides, to chemically anchor LiPSs and mitigate the shuttle effect;^[^ ^15^ ^]^ 3) utilizing electrocatalysts to accelerate redox kinetics and improve the overall efficiency of Li–S chemistry.^[^ ^16^ ^]^ Among the numerous options for such materials, metal sulfides have emerged as promising candidates, as they not only effectively immobilize soluble LiPSs through strong polar–polar interactions but also exhibit excellent catalytic activity for the necessary redox reactions involving sulfur and polysulfides. Molybdenum disulfide (MoS_2_), a typical metal sulfide, has been widely applied as the host material in Li−S batteries due to its moderate polar interaction with LiPSs and its ability to facilitate fast Li^+^ diffusion. Recent studies have clearly highlighted the advances of MoS_2_‐based materials in Li−S batteries. For instance, Jin et al.^[17a]^ employed a Zn^2+^ intercalation strategy to induce the phase transition from semiconducting 2H to metallic 1T MoS_2_, thereby improving the electrocatalytic performance for the conversion and decomposition of LiPSs. Zhang et al.^[17b]^ developed a stable 1T‐MoS_2_/carbon composite via 3D printing, which strengthened LiPSs binding and reduced Li_2_S nucleation barriers. Bai et al.^[17c]^ dispersed Ru single atoms on MoS_2_/MXene, creating synergistic “adsorption−catalysis” interfaces that accelerated LiPS conversion and effectively suppressed LiPSs shuttling. The resulting Ru−MoS_2_/MXene/S cathode delivered a high discharge capacity of 726 mAh g^−1^ at a sulfur loading of 9.5 mg cm^−2^ with a lean electrolyte‐to‐sulfur ratio of 4.3. These advances demonstrate the versatility of MoS_2_ as a LiPS‐trapping and catalytic component, enabling improved redox kinetics and cycling stability under practical cell conditions. To further decrease polarization and stabilize nanosized MoS_2_ particles, these materials are often integrated with conductive carbons alongside sulfur. Hence, the combination of MoS_2_ and lignin‐derived carbon materials would be a suitable option to design advanced sulfur hosts. However, to date, there have been no reports on embedding metal sulfides in lignin‐derived CNFs for the application in Li−S batteries. Notably, most reported carbon‐based host materials for Li−S batteries exist in powder form, necessitating the addition of conductive carbon and binders to prepare slurries, which are subsequently coated onto metallic current collectors.^[^ ^18^ ^]^ The incorporation of binders in electrode fabrication introduces structural instabilities, resulting in electrode cracking, active material detachment, side reactions, and disruption of conductivity, all of which contribute to more or less rapid capacity fading.^[^ ^19^ ^]^ Taking these considerations into account, electrospun CNFs seem to be attractive materials due to their high porosity, large surface area, and excellent electrical conductivity. These characteristics enable them to simultaneously serve as both a sulfur host and a current collector, offering a lightweight, binder‐free alternative to powdered carbons for high‐performance Li–S batteries.^[^ ^20^ ^]^

Herein, a sustainable and biomass‐derived strategy is presented for fabricating free‐standing CNFs embedded with catalytic MoS_2_ nanoparticles, derived from lignin via electrospinning and subsequent pyrolysis in an inert atmosphere. The lightweight MoS_2_@CNFs film with interconnected 1D CNFs networks simultaneously functions as the current collector and sulfur host, eliminating the need for additional conductive carbon and binders. Additionally, MoS_2_ nanoparticles chemically interact with LiPSs to suppress their dissolution and migration to the Li anode, thus mitigating the shuttle effect. Moreover, MoS_2_ nanoparticles could serve as electrocatalysts to accelerate sluggish solid–liquid–solid conversion reaction kinetics. With these synergistic advantages, Li–S batteries with MoS_2_@CNFs‐based cathode exhibit excellent rate performance (519.6 mAh g^−1^ at 2C) and cycling stability (609.3 mAh g^−1^ after 200 cycles at 1C). This work offers an efficient strategy for converting lignin into high‐value CNFs film, providing a sustainable and scalable approach for binder‐free electrode fabrication in energy storage applications.

## 2 Results and Discussion

**Figure 1:**
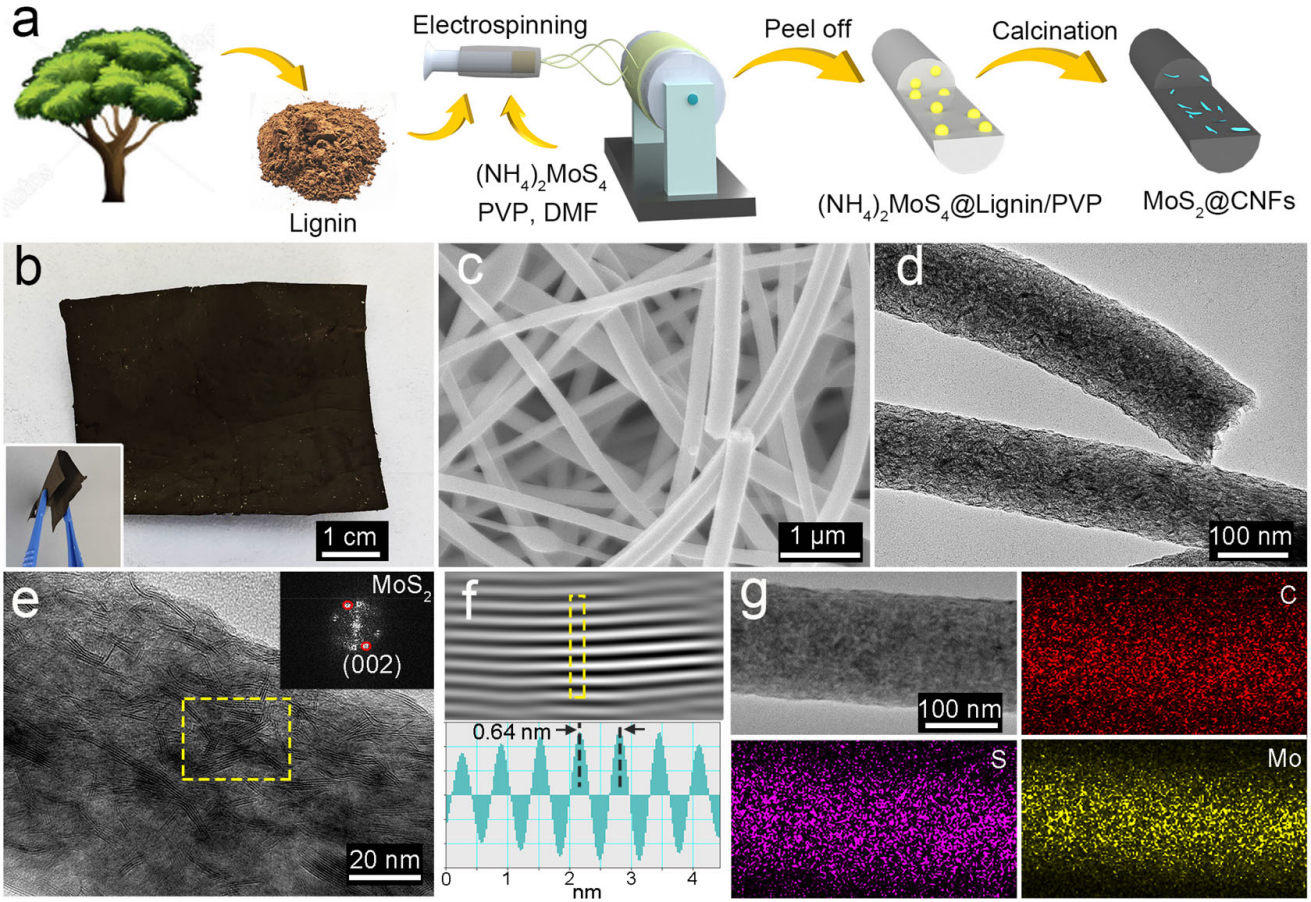
a) Schematic illustration of the preparation for the MoS_2_/CNFs from lignin. b) Digital image of the free‐standing MoS_2_/CNFs. Inset: bending test of the MoS_2_/CNFs membrane. c) SEM image, d) TEM image, and e) HRTEM image of the MoS_2_/CNFs. f) Line profile for the selected line in its inverse FFT image. g) EDS elemental mapping of C, S, and Mo in the MoS_2_/CNFs.

The MoS_2_/CNFs film was synthesized by electrospinning, followed by carbonization in an argon atmosphere, as illustrated in **Figure** **1**a. Direct electrospinning of a pure lignin solution is challenging, as it primarily yields lignin nanospheres rather than continuous fibers (Figure S1, Supporting Information). This limitation arises from the inherently short molecular chains, low flexibility, complex macromolecular structure, and irregular molecular arrangement of lignin, all of which hinder its spinnability during electrospinning.^[^ ^21^ ^]^ The common way to improve the spinnability of lignin is the addition of long‐chain polymers to improve the molecular chain entanglement. Even the addition of just 1 wt% polyethylene oxide (M_w_ ≈ 1 × 10^6^ g mol^−1^) to the lignin solution significantly enhances its electrospinnability, enabling the formation of continuous nanofibers.^[^ ^22^ ^]^ In this work, lignin and 5 wt% polyvinylpyrrolidone (PVP, M_w_ = 1300 kDa) were dissolved in dimethylformamide (DMF) to prepare the electrospinning solution. This solution was loaded into a syringe with a needle and connected to a pump to control the flow rate. The distance between the needle and collector was 15 cm, and the electrospinning process was conducted at a constant flow rate of 2 mL h^−1^ under a voltage of 12 kV. Ammonium tetrathiomolybdate ((NH_4_)_2_MoS_4_) was added as the MoS_2_ precursor, which has been widely utilized in Li−S batteries due to its strong adsorption of lithium polysulfide and excellent catalytic activity for the involved redox reactions.^[^ ^23^ ^]^ After electrospinning, the polymer nanofibers were first peroxided at 250 °C for 2 h in the air and then calcinated at 800 °C with a ramp of 5 °C min^−1^ under a flow Ar atmosphere and maintained for 2 h to get free‐standing MoS_2_/CNFs (Figure 1b).

The morphology of the obtained polymer nanofibers and MoS_2_/CNFs was examined using scanning electron microscopy (SEM) and transmission electron microscopy (TEM). The as‐prepared ((NH_4_)_2_MoS_4_)@lignin/PVP nanofibers formed free‐standing fabrics with intertwined fiber morphology, as shown in Figure S2, Supporting Information, confirming the successful synthesis of ((NH_4_)_2_MoS_4_)@lignin/PVP nanofibers. After pyrolysis at 800 °C under argon, the ((NH_4_)_2_MoS_4_)@lignin/PVP nanofibers were converted to CNFs embedded with MoS_2_ nanoparticles. As seen in the inset in Figure 1b, the MoS_2_/CNFs maintain a free‐standing structure, allowing them to be directly used as the host materials for Li−S batteries, eliminating the need for additional conductive carbon and binders. The SEM image of MoS_2_/CNFs (Figure 1c) shows a well‐preserved fiber morphology with a diameter of 214.0 ± 46.1 nm (Figure S3, Supporting Information). High‐resolution TEM (HRTEM) images of the MoS_2_/CNFs reveal numerous MoS_2_ nanoparticles with a layered structure, which are homogeneously embedded within the CNFs (Figure 1d,e). The control sample was prepared using the same method as MoS_2_/CNFs but without (NH_4_)_2_MoS_4_, resulting in pure CNFs. The TEM image of the CNFs is shown in Figure S4, Supporting Information. The diameter of pure CNFs is significantly larger than that of MoS_2_/CNFs, likely due to the addition of ammonium tetrathiomolybdate, which alters the viscosity, conductivity, and surface tension of the electrospinning solution. These changes result in the formation of thinner jets during electrospinning, leading to smaller fiber diameters after carbonization.^[^ ^24^ ^]^ Unlike CNFs, MoS_2_/CNFs exhibit a porous structure characterized by irregularly shaped pores, which are formed due to gas evolution, primarily hydrogen sulfide and ammonia, during the pyrolysis of (NH_4_)_2_MoS_4_.^[^ ^25^ ^]^ The inverse fast Fourier transform (FFT) image's line profile confirms a d‐spacing of 6.4 Å for MoS_2_ nanoparticles, corresponding to the (002) plane of hexagonal MoS_2_ (Figure 1f).^[^ ^26^ ^]^ The uniform distribution of MoS_2_ nanoparticles within the MoS_2_/CNFs is further corroborated by energy‐dispersive X‐ray spectrometry (EDS) images (Figure 1g), which show evenly distributed Mo and S signals throughout the C signal.

**Figure 2:**
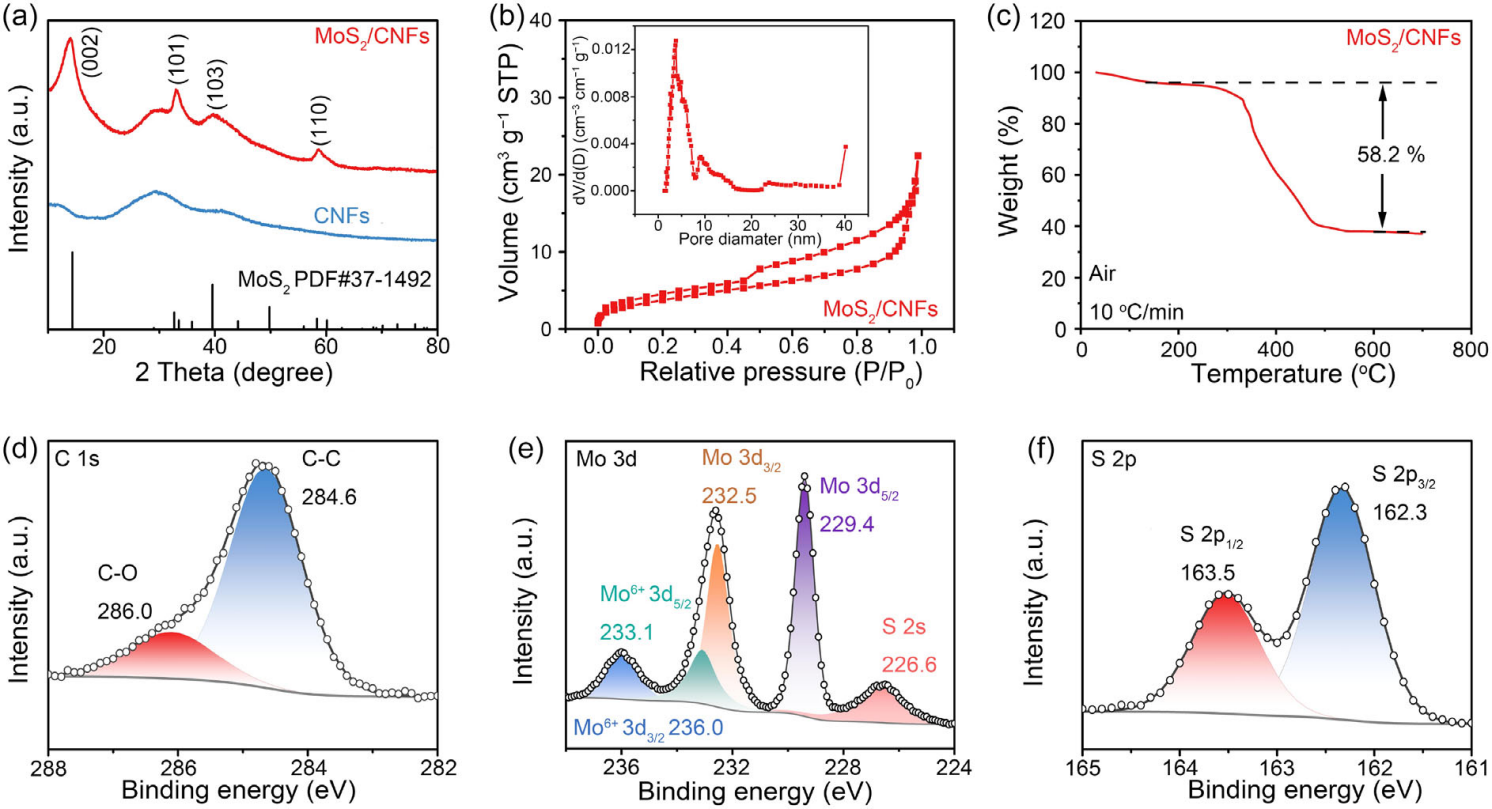
a) XRD patterns of the MoS_2_/CNFs and CNFs. b) Nitrogen adsorption−desorption isotherms of the MoS_2_/CNFs. Inset: the corresponding pore size distribution plot of the MoS_2_/CNFs. c) TGA curve of the MoS_2_/CNFs under air from room temperature to 700 °C at a ramping rate of 10.0 °C min^−1^. XPS spectra of the C 1s d), Mo 3d e), and S 2p f) of the MoS_2_/CNFs.

The chemical composition of MoS_2_/CNFs and CNFs was analyzed using X‐ray diffraction (XRD) and X‐ray photoelectron spectroscopy (XPS). The XRD pattern of CNFs shows a broad reflex at 2 Theta of ≈29.4° (**Figure** **2**a), corresponding to the stacking of the slightly ordered carbon.^[^ ^27^ ^]^ In the XRD pattern of MoS_2_/CNFs, distinct peaks are observed at 2θ of 14.2°, 33.1°, 39.5°, and 58.8°, which are indexed to the (002), (101), (103), and (110) lattice planes of hexagonal MoS_2_, respectively.^[^ ^28^ ^]^ The absence of additional peaks in the XRD pattern of MoS_2_/CNFs confirms the complete conversion of (NH_4_)_2_MoS_4_ into MoS_2_ during high‐temperature calcination without any impurities. The specific surface area and pore structure of MoS_2_/CNFs were further analyzed using nitrogen adsorption–desorption isotherms (Figure 2b). The MoS_2_/CNFs exhibit a type IV isotherm, with a specific surface area of 38.9 m^2^ g^−1^, higher than that of CNFs (27.2 m^2^ g^−1^, Figure S5, Supporting Information). The pore size distribution of MoS_2_/CNFs is centered around 5 nm.[^13^, ^29^] These pores, generated by the gas produced from pyrolysis of (NH_4_)_2_MoS_4_, as seen in the TEM images shown in Figure 1d, can facilitate sulfur accommodation and electrolyte penetration.^[^ ^30^ ^]^ The MoS_2_ content in the MoS_2_/CNFs was determined through thermogravimetric analysis (TGA), as shown in Figure 2c. An initial weight loss of 5 % occurs below 100 °C, attributed to the evaporation of adsorbed moisture. A more significant weight loss, starting around 350 °C and accounting for 58.2% of the total mass, results from the oxidation of carbon and MoS_2_ in air. The initial MoS_2_ content in the MoS_2_/CNFs is estimated to be 43.4 wt%, based on the MoO_3_ residual.^[^ ^31^ ^]^ XPS spectra, as shown in Figure 2d–f, provide detailed information on the surface composition and chemical states of the MoS_2_/CNFs. In the high‐resolution C 1s XPS spectrum (Figure 2d), two distinct peaks appear at 284.6 eV and 286.0 eV, corresponding to graphitic sp^2^ hybridized carbon and C—O bonds, respectively.^[^ ^32^ ^]^ The Mo 3d XPS spectrum (Figure 2e) displays two peaks at 229.4 eV and 232.5 eV, which are assigned to Mo 3d_5/2_ and Mo 3d_3/2_, characteristic of Mo^4+^ in MoS_2_/CNFs.^[^ ^33^ ^]^ Additionally, a pair of peaks at 233.1 eV and 236.0 eV is attributed to Mo^6+^, caused by surface oxidation of the MoS_2_ layer in air.^[^ ^34^ ^]^ The peak at 226.6 eV corresponds to the S 2s component in MoS_2_.^[^ ^35^ ^]^ In the S 2p XPS spectrum (Figure 2f), peaks at 163.5 eV (S 2p_1/2_) and 162.3 eV (S 2p_3/2_) originate from divalent sulfide ions S^2−^ in MoS_2_.^[^ ^36^ ^]^ These measurements confirm the successful synthesis of MoS_2_/CNFs.

**Figure 3:**
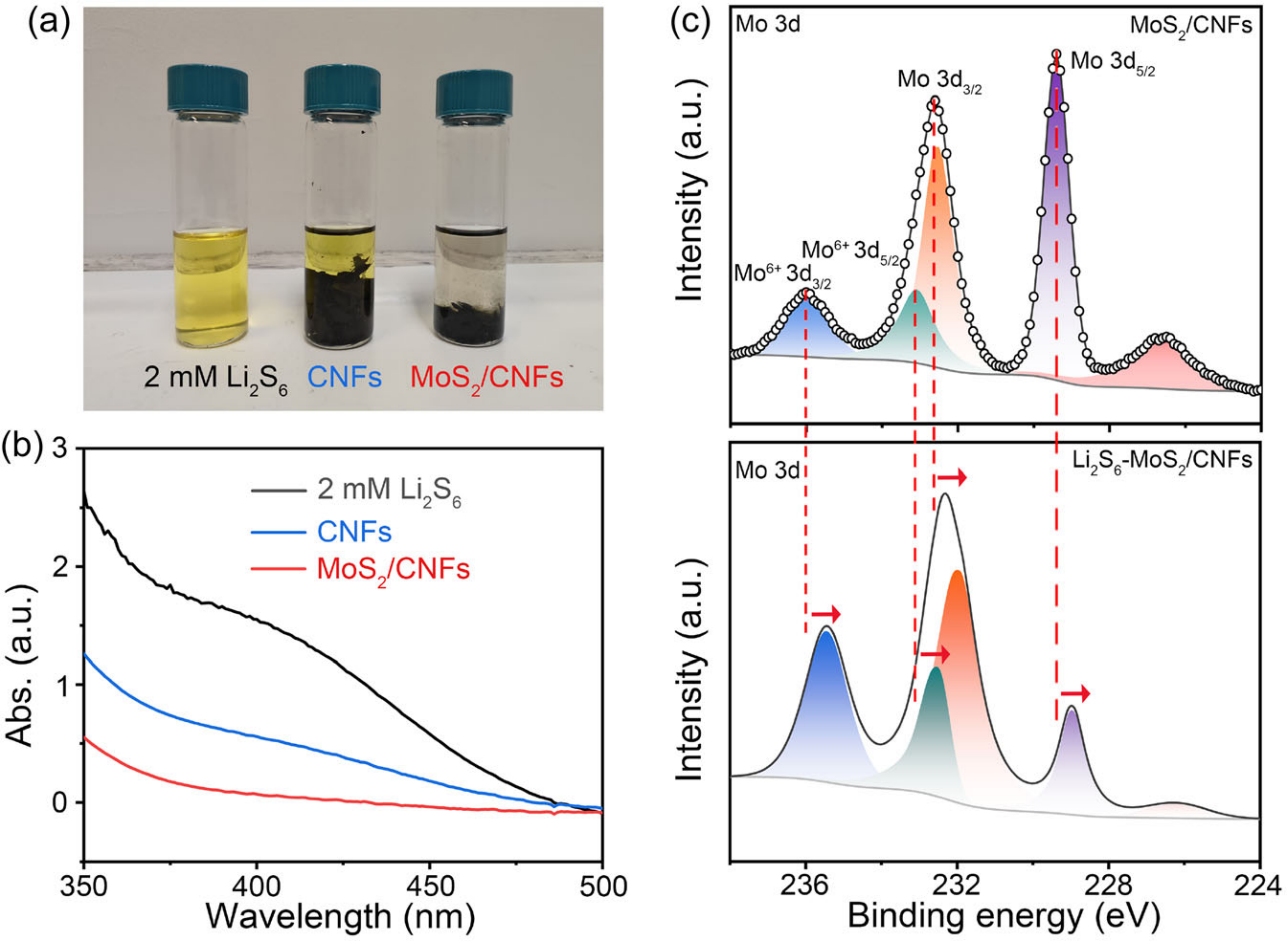
a) Digital image of the 4 mL 2.0 mM Li_2_S_6_ electrolyte after mixing with 20 mg MoS_2_/CNFs or CNFs for 3 h. b) UV–vis absorption spectra of the Li_2_S_6_ electrolyte after mixing with MoS_2_/CNFs and CNFs for 3 h. c) Mo 3d XPS spectra of MoS_2_/CNFs before (top) and after (below) Li_2_S_6_ adsorption test.

Li−S batteries undergo complex multiphase and multielectron reactions in the sulfur cathode, involving solid S_8_, dissolved lithium polysulfides (LiPSs), and solid Li_2_S.^[^ ^37^ ^]^ Driven by the strong electric field and concentration gradient between the cathode and anode, soluble LiPSs tend to diffuse toward the Li anode, causing the shuttle effect and capacity degradation.^[^ ^38^ ^]^ To mitigate this issue, the development of polar sulfur host materials with strong LiPSs anchoring capabilities has been widely employed. To explore the interactions between different host materials and polysulfide species, a visual adsorption test was conducted. As depicted in **Figure** **3**a, an equal mass of MoS_2_/CNFs and CNFs (20 mg) was immersed in 4 mL 2 mM yellow Li_2_S_6_ solution (solvent: DOL/DME, v/v = 1:1). After standing for 3 h, the yellow color of the Li_2_S_6_ solution decays with different degrees in the presence of MoS_2_/CNFs and CNFs. The color of the Li_2_S_6_ solution containing MoS_2_/CNFs is lighter than that of the solution containing CNFs, indicating the strong adsorption ability of MoS_2_/CNFs for polysulfides. The ultraviolet−visible (UV−vis) absorption spectra of the supernatant of polysulfide solutions adsorbed by MoS_2_/CNFs and CNFs are presented in Figure 3b. The lowest absorbance of the polysulfide solutions adsorbed by MoS_2_/CNFs in the wavelength range of 350–500 nm further verifies the strong affinity between MoS_2_/CNFs and soluble polysulfides. The chemical interaction between different host materials and Li_2_S_6_ has been explored by XPS before and after Li_2_S_6_ adsorption tests (Figure 3c). Compared with the Mo 3d spectra of the MoS_2_/CNFs before adsorption (229.4, 232.5, 233.1, and 236.0 eV for Mo 3d_5/2_, Mo 3d_3/2_, Mo^6+^ 3d_5/2_ and Mo^6+^ 3d_3/2_, respectively), the binding energies of Mo species shift to lower values (228.9, 231.9, 232.5 eV, and 235.4 eV for Mo 3d_5/2_, Mo 3d_3/2_, Mo^6+^ 3d_5/2_ and Mo^6+^ 3d_3/2_, respectively). The C 1s spectra of MoS_2_/CNFs before and after Li_2_S_6_ adsorption show no shift (Figure S6, Supporting Information), indicating that the observed shift in the Mo 3d spectra is due to surface changes rather than measurement artifacts. These results are consistent with previous reports,^[^ ^39^ ^]^ which demonstrate that polar Mo sites in MoS_2_ can strongly anchor polysulfides through Mo−S/Li−S interactions. The unsaturated Mo atoms serve as Lewis acidic centers, effectively binding with the S atoms in LiPSs, while the S sites in MoS_2_ provide additional anchoring capability, thereby suppressing the polysulfide shuttle.

**Figure 4:**
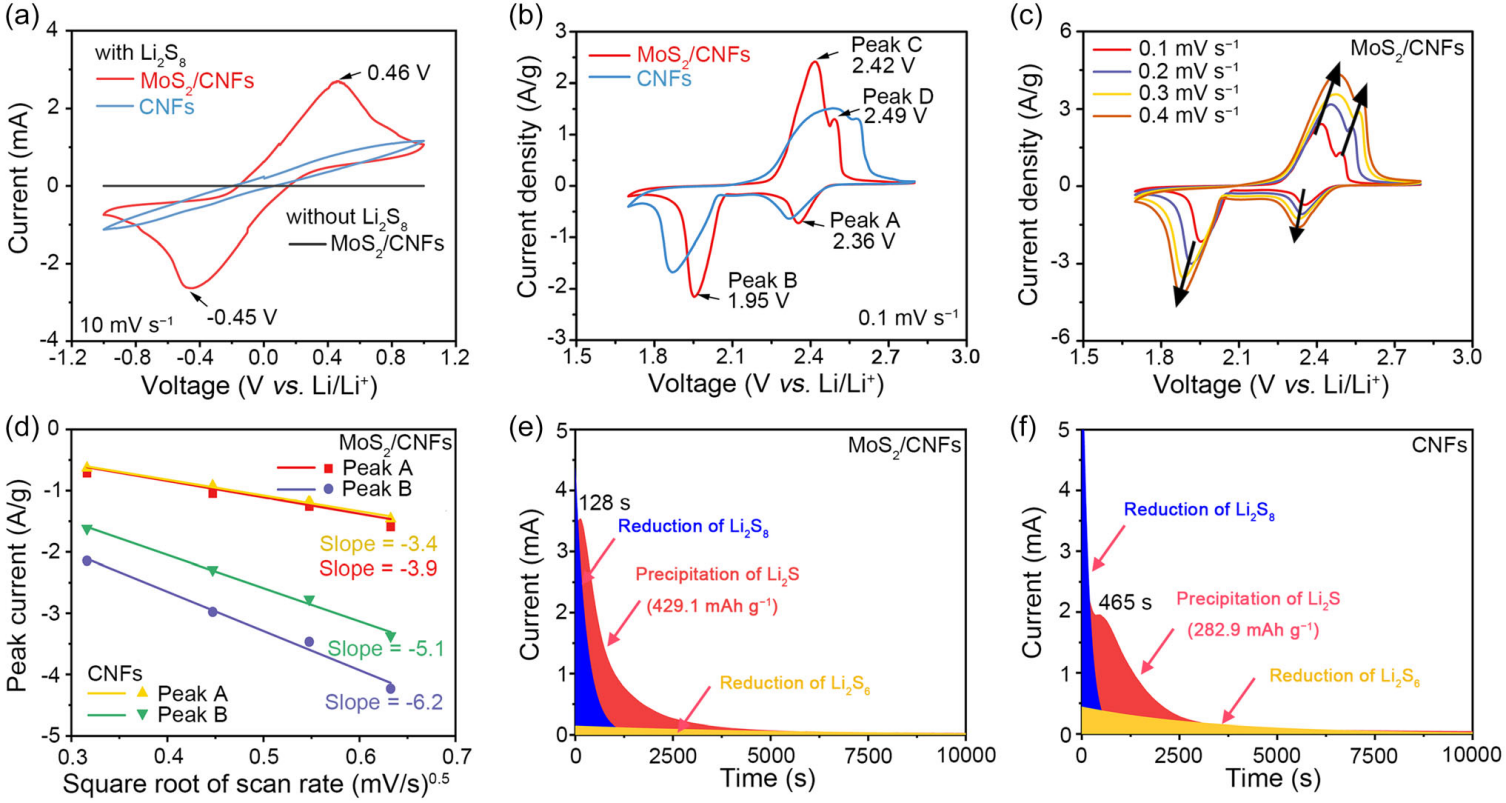
a) CV curves of the Li_2_S_8_ symmetric cell with MoS_2_/CNFs and CNFs electrodes with a scan rate of 10 mV s^−1^ in the voltage range of −1.0 V to 1.0 V. b) CV curves of the coin cell with the MoS_2_/CNFs and CNFs‐based cathodes in the voltage range of 1.7 to 2.8 V at a scan rate of 0.1 mV s^−1^. c) CV curves of the coin cell with the MoS_2_/CNFs electrodes in the voltage range of 1.7 to 2.8 V at various scan rates of 0.1, 0.2, 0.3, and 0.4 mV s^−1^. d) Plot of CV peak current of the peak A (S_8_ → Li_2_S_4_) and peak B (Li_2_S_4_ → Li_2_S) versus the square root of scan rate. Current‐time plots of Li_2_S_8_ catholyte potentiostatically discharged at 2.05 V on e) MoS_2_/CNFs‐based cathode and f) CNFs‐based cathode.

Notably, the amphipathic LiPS‐trapping (sulfiphilicity and lithiophilicity) capabilities are propitious to accelerate charge transfer between MoS_2_/CNFs hosts and LiPS guests, thus promoting redox reactions of Li−S electrochemistry, as verified by the symmetrical cell measurement (**Figure** **4**a). A 0.5 m Li_2_S_8_ solution was used as a catholyte to assemble symmetrical cells with the identical working electrode and counter electrode containing either MoS_2_/CNFs or CNFs electrodes. The cyclic voltammetry (CV) curves of these symmetrical cells between −1.0 and 1.0 V at a scan rate of 10 mV s^−1^ are shown in Figure 4a. The symmetrical cell without Li_2_S_8_ solution exhibited no capacity, indicating that double‐layer capacitance did not contribute to the overall current response. For the CV curve of the symmetrical cell with MoS_2_/CNFs electrode, a pair of peaks centered at −0.45 (cathodic) and 0.46 V (anodic) were detected, while the symmetrical cell with CNFs electrode displays no distinguishable redox peaks and weak current, suggesting that the chemical interaction between MoS_2_ and LiPSs not only statically exists but also dynamically accelerates the electrochemical reactions of LiPSs.

To investigate the properties of the MoS_2_/CNFs as sulfur hosts in Li−S chemistry, the electrochemical performances of the MoS_2_/CNFs and CNFs‐based cathodes were examined in Li–S coin cells with a sulfur loading of 1 mg cm^−2^ (0.5 m Li_2_S_8_ catholyte) and electrolyte‐to‐sulfur (E/S) ratio of 32 μL mg^−1^. The CV curves of the MoS_2_/CNFs and CNFs‐based cathodes were measured in the voltage window of 1.7−2.8 V versus Li/Li^+^ at a scan rate of 0.1 mV s^−1^, as presented in Figure 4b. The CV curve of the MoS_2_/CNFs‐based cathode shows two reductive peaks (peak A and peak B) at 2.36 and 1.95 V, corresponding to the reduction of S_8_ to high‐order LiPSs (Li_2_S_x_, 4 ≤ × ≤ 8) and then to low‐order Li_2_S_2_/Li_2_S, respectively, and two oxidation peaks (peak C and peak D) at 2.42 and 2.49 V, corresponding to the oxidation of low‐order Li_2_S_2_/Li_2_S to high‐order LiPSs and further to S_8_, respectively. The peak position and peak current density of MoS_2_/CNFs and CNFs‐based cathodes were summarized in Table S1, Supporting Information. Compared with CNFs, the MoS_2_/CNFs‐based electrode shows positive shifts of peak A/B by 20−80 mV and negative shifts of C/D by ≈70 mV, reducing the overall peak separation (the voltage difference between peak B and peak C) from ≈0.62 to ≈0.47 V. Moreover, the MoS_2_/CNFs‐based electrode shows higher peak current density than that of the CNFs‐based electrode. This comparison indicates the lower polarization of MoS_2_/CNFs‐based electrodes and the strong catalytic effect of MoS_2_ nanoparticles toward sulfur redox processes. To obtain further insight into the role of MoS_2_/CNFs‐based cathode in accelerating the LiPSs conversion reaction, the CV tests were performed at different scan rates from 0.1 to 0.4 mV s^−1^ (Figure 4c and Figure S7a, Supporting Information) to characterize the diffusion coefficient of Li^+^ (*D*_Li_) through the Randles–Sevcik equation.^[13b]^ When increasing the scan rate, the cathodic peaks shifted to more negative potentials and the anodic peak shifted to a more positive potential, overall increasing the polarization voltage. The linear relationship between the anodic/cathodic peak current and the square root of the scanning rates pointed to a diffusion‐limited reaction (Figure 4d and Figure S7b, Supporting Information). Thus, the classical Randles−Sevcik equation was used to calculate the Li^+^ diffusion coefficient (1):^[13b]^

(1) $$I_{p}=\left(2.69\;\times 10^{5}\right)\;n^{1.5}A\;D_{\text{Li}}^{1.5}\;C_{\text{Li}}\;v^{0.5}$$

where *I*_*p*_ is the peak current, *n* is the number of charge transfers, *A* is the geometric electrode area, *D*_Li_ is the Li^+^ diffusion coefficient, *C*_Li_ is the concentration of Li^+^ in the electrolyte, and *ν* is the scan rate. In this case, *n*, *A*, and C_Li_ can be regarded as constant; sharper I_p/_ *ν* ^0.5^ slopes denote faster Li^+^ diffusion. As displayed in Figure 4d and Figure S7b, Supporting Information, the MoS_2_/CNFs‐based cathode exhibits the sharpest slopes for all the oxidation and reduction peaks, indicating the highest Li^+^ diffusivity during the redox reactions. Generally, rapid Li^+^ diffusion promotes the transformation of sulfur species in the cathode, indicating the enhanced reaction kinetics.^[^ ^40^ ^]^

Ideally, the conversion reaction from liquid Li_2_S_4_ to solid Li_2_S accounts for 75% of the theoretical capacity and encompasses the most kinetically sluggish solid‐phase conversion.^[^ ^41^ ^]^ Therefore, a facilitated Li_2_S precipitation process could play a pivotal role in enhancing the electrochemical performance of Li–S batteries. The Li_2_S precipitation tests were conducted by using Li_2_S_8_ catholyte to study the effects of the MoS_2_ particles on the liquid–solid conversion processes from LiPSs to Li_2_S during cycling.^[13b]^ After the galvanostatic discharging step, the coin cells were potentiostatically discharged at 2.05 V to initiate the deposition of Li_2_S. Time‐dependent reduction current curves of the cells with MoS_2_/CNFs and CNFs‐based electrodes were collected and displayed in Figure 4e and f, respectively. The MoS_2_/CNFs‐based electrode achieves a higher current of 3.53 mA after 128 s (Figure 4e), compared to the CNFs‐based electrode, which reaches 1.96 mA after 465 s (Figure 4f). Moreover, the MoS_2_/CNFs‐based electrode exhibits a higher Li_2_S precipitation capacity of 429.1 mAh g^−1^ compared to 282.9 mAh g^−1^ for the CNFs‐based electrode, indicating the improved conversion kinetics from LiPS to Li_2_S for the MoS_2_/CNFs‐based electrode.

**Figure 5:**
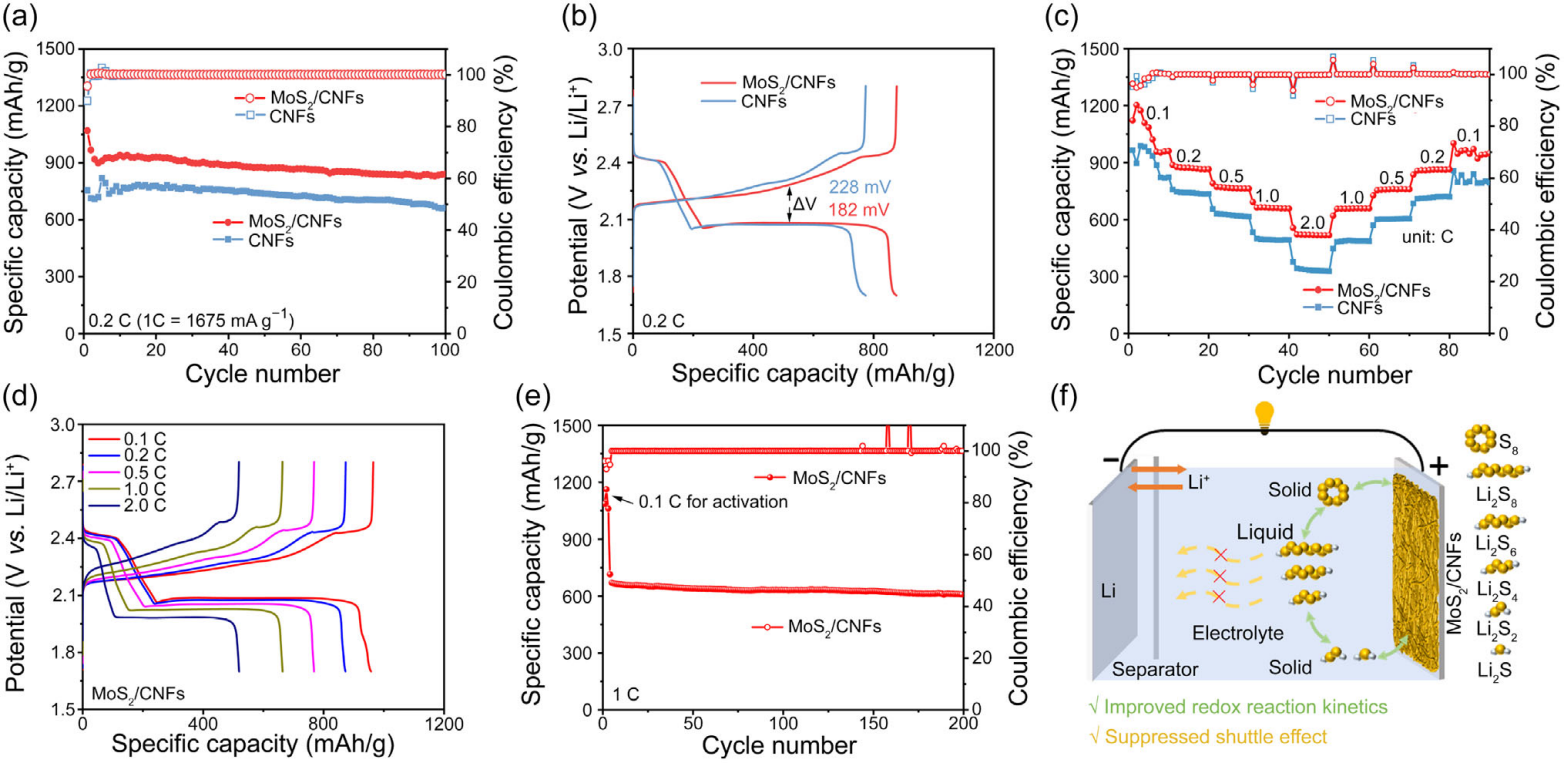
a) Cycling performance and b) charging/discharging curve profiles of Li−S cells based on MoS_2_/CNFs and CNFs‐based cathodes at 0.2C. c) Rate performance and d) charge/discharge curve profiles of Li−S cells based on MoS_2_/CNFs and CNFs‐based cathodes at various rates from 0.1 to 2C. e) Long‐cycle performance of the Li−S cells based on MoS_2_/CNFs‐based cathodes at 1C. The sulfur loading is 1 mg cm^−2^ (0.5 m Li_2_S_8_ catholyte) and the electrolyte‐to‐sulfur (E/S) ratio is 32 μL mg^−1^. f) Schematic illustration of the advantages of the MoS_2_/CNFs host materials in Li−S batteries.

A series of catalytic activity evaluations has clarified the enhanced conversion kinetics driven by MoS_2_/CNFs for the Li–S system. To confirm this, the electrochemical performances of the MoS_2_/CNFs and CNFs‐based electrodes were evaluated in Li−S coin cells. The free‐standing MoS_2_@CNFs were directly used as the host material without the need for additional conductive carbon and binder. **Figure** **5**a presents the cycling performance of the MoS_2_/CNFs and CNFs‐based cathodes at a current density of 0.2C (1 C = 1675 mA g^−1^) for 100 cycles. MoS_2_/CNFs‐based cathode delivered an initial discharge capacity of 1069.7 mAh g^−1^ at 0.2C, which was retained at 840.5 mAh g^−1^ after 100 cycles. In contrast, the CNFs‐based cathode delivered a lower initial discharge capacity of 755.5 mAh g^−1^ at the same current density, retaining 664.4 mAh g^−1^ after 100 cycles. The higher discharge capacity of the MoS_2_/CNFs‐based cathode is mainly due to the strong LiPSs adsorption ability of MoS_2_ nanoparticles that can chemically adsorb LiPSs and improve active material utilization. The galvanostatic charge–discharge (GCD) curves of MoS_2_/CNFs and CNFs‐based cathodes at 0.2C are presented in Figure 5b. Both GCD curves show two discharge platforms, located at around 2.4 V and 2.1 V, corresponding to the 4‐electron reduction of sulfur to soluble long‐chain LiPSs (S_8_ + 4Li^+^ + 4e^−^ → 2Li_2_S_4_) and the subsequent 12‐electron reaction to insoluble lithium sulfide (2Li_2_S_4_ + 12Li^+^ + 12e^−^ → 8Li_2_S).^[^ ^38^ ^]^ The anodic plateau, located at around 2.2 and 2.3 V, obtained during the charging process, is attributed to a reverse multistep sulfur oxidation process, in which short‐chain sulfides are converted to LiPSs and eventually to sulfur, respectively. The overpotential between the charge and discharge curves at half‐discharge capacity for the MoS_2_/CNFs‐based cathode (182 mV) is lower than that for the CNFs‐based cathode (228 mV), as corroborated by the CV measurements in Figure 4b. This result confirms the accelerated redox reaction in the MoS_2_/CNFs‐based cathode, due to the strong chemical adsorption of LiPSs by MoS_2_ nanoparticles and the rapid Li^+^ diffusion.

The rate performance of MoS_2_/CNFs and CNFs‐based cathodes was assessed at various current densities from 0.1 to 2C. As shown in Figure 5c, the MoS_2_/CNFs‐based cathode delivered specific discharge capacities of 1085.4, 872.6, 766.5, 661.8, and 519.6 mAh g^−1^ at current densities of 0.1, 0.2, 0.5, 1C, and 2C, respectively. However, due to the severe polysulfide shuttle and sluggish conversion reaction kinetics, the CNFs‐based cathode delivered a much lower specific discharge capacity of 960.4, 742.4, 624.5, 494.2, and 332.4 mAh g^−1^ at 0.1, 0.2, 0.5, 1C, and 2C, respectively. Besides, the MoS_2_/CNFs‐based cathode recovered an average capacity of 966.7 mAh g^−1^ when the current rate was returned to 0.1C, higher than that of the CNFs‐based cathode, which only retained at 800.3 mAh g^−1^. Figure 5d displays the GCD curves of the Li−S batteries with MoS_2_/CNFs‐based cathode at different current rates. All discharge curves of the MoS_2_/CNFs‐based cathode show two discharge plateaus, even at 2C, and show lower polarization voltage compared to the CNFs‐based cathode (Figure S8, Supporting Information). The long‐cycling performance of the MoS_2_/CNFs‐based cathode was measured for 200 cycles at 1C (Figure 5e). After initial activation at 0.1C for 3 cycles, the MoS_2_/CNFs‐based cathode delivered a specific discharge capacity of 715.3 mAh g^−1^ at 1C and maintained at 609.3 mAh g^−1^ after 200 cycles, corresponding to a capacity decay of 0.074% per cycle. Table S2, Supporting Information, compares the electrochemical performance of the MoS_2_/CNFs‐based electrode with other lignin/biomass and MoS_2_‐based hosts reported in the literature. Our free‐standing MoS_2_/CNFs electrode delivers a comparable and even better specific discharge capacity compared to other lignin/biomass and MoS_2_‐based hosts. This comparison highlights the effectiveness of our strategy for converting waste lignin into high‐performance, multifunctional electrode materials for Li−S batteries.

**Figure 6:**
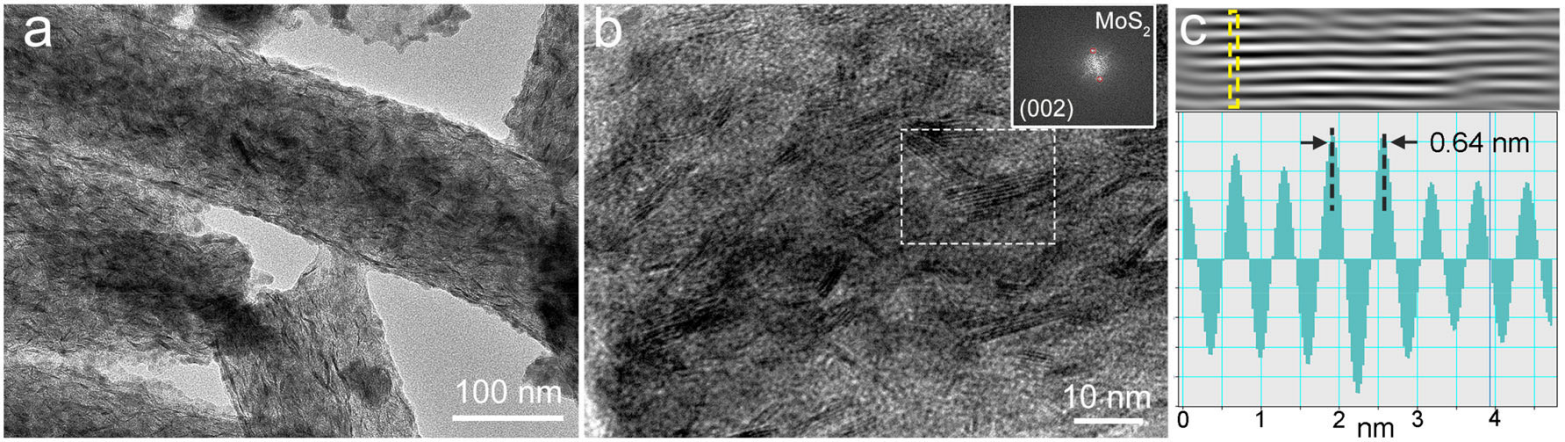
a) TEM image of the MoS_2_/CNFs after cycling at 1C for 200 cycles. b) HRTEM image of the MoS_2_/CNFs after cycling at 1C for 200 cycles. Inset: FFT filtered image derived from the white box area in the HRTEM image. c) Line profile for the selected line in its inverse FFT image.

After 200 cycles at 1C, the coin cell was disassembled, and the MoS_2_/CNFs‐based cathode was taken out for TEM measurement. As shown in **Figure** **6**a–b, the layered MoS_2_ nanoparticles are clearly observed in the CNF after cycling. The HRTEM images display a d‐spacing of 6.4 Å, which can be indexed to the (002) plane of hexagonal MoS_2_ (Figure 6c). This is consistent with the pristine MoS_2_ nanosheets shown in Figure 1f, demonstrating their excellent structural integrity. Electrochemical impedance spectroscopy of the Li−S batteries with the MoS_2_/CNFs and CNFs‐based electrode was measured before cycling, as shown in Figure S9, Supporting Information. The Nyquist plot shows a semicircle in the high‐frequency region denoting the charge‐transfer process at the interface, while a linear section in the low‐frequency region represents lithium diffusion within the electrode. Both electrodes exhibit comparable high‐frequency intercepts, indicating similar solution resistance (*Rs*). The MoS_2_/CNFs‐based cathode shows a smaller charge‐transfer resistance (*Rct*) than the CNFs counterpart (83.5 and 94.7 Ω for the MoS_2_/CNFs and CNFs‐based cathodes, respectively). These features corroborate that polar/catalytic MoS_2_ anchored on conductive CNFs scaffold accelerates interfacial kinetics and ion diffusion, accounting for the improved rate capability and cycling stability.

The improved electrochemical performance of the MoS_2_/CNFs‐based cathode compared to the CNFs‐based cathode is primarily attributed to the synergistic integration of catalytic functionality and a self‐supporting 3D architecture. The embedded polar MoS_2_ nanoparticles serve as catalytic sites that chemically anchor intermediates LiPSs, effectively suppressing the shuttle effect and accelerating sulfur redox kinetics. Simultaneously, the free‐standing nanofibrous framework, derived from lignin, ensures robust structural integrity and efficient electron/ion transport pathways without the need for a binder or conductive additive. These advantages are illustrated in Figure S11, Supporting Information.

## 3 Conclusion

In summary, we synthesized free‐standing MoS_2_ nanoparticle‐embedded CNFs (MoS_2_@CNFs) using lignin as a sustainable carbon precursor via electrospinning and subsequent pyrolysis. By incorporating 5 wt% PVP and (NH_4_)_2_MoS_4_ into the lignin solution, uniform lignin/PVP nanofiber films were fabricated. After preoxidation and calcination, CNFs with an average diameter of 214.0 ± 46.1 nm were obtained, which were embedded with well‐dispersed MoS_2_ nanoparticles. The resulting MoS_2_@CNFs cathode host leveraged the synergistic effect of strong polysulfide adsorption and electrocatalytic activity from the embedded MoS_2_, as well as the robust, binder‐free 3D architecture of the carbon framework. As a result, it demonstrated excellent rate performance (519.6 mAh g^−1^ at 2C) and cycling stability (609.3 mAh g^−1^ after 200 cycles at 1C). This work highlights a sustainable and scalable strategy for converting waste lignin into high‐performance, multifunctional electrode materials for Li−S batteries, with potential applicability to other energy storage systems.

## 4 Experimental Section

The experimental details are provided in the Supporting Information (SI). This section briefly summarizes the synthesis and measurements. The MoS_2_/CNFs were prepared by using the electrospinning method (Spinbox System, Bioinicia S.L.). The electrospinning solution was prepared by adding 3.8 g Lignin, 0.2 g polyvinyl pyrrolidone (PVP, average Mw = 1 300 000), and 0.5 g (NH_4_)_2_MoS_4_ into 10 mL dimethylformamide and stirring for 12 h. This solution was loaded into a syringe with a needle and connected to a pump to control the flow rate. The distance between the needle and collector was 15 cm, and the electrospinning process was conducted at a constant flow rate of 2 mL h^−1^ under a voltage of 12 kV. Subsequently, the collected polymer nanofibers were peroxidized at 250 °C for 2 h in air. Then, the sample was heated to 800 °C with a ramp of 5 °C min^−1^ under a flow of Ar atmosphere and maintained for 2 h to prepare the MoS_2_/CNFs. The pure CNFs were prepared using the same method as MoS_2_/CNFs, but without adding (NH_4_)_2_MoS_4_.

The morphology of the obtained samples was investigated by a LEO 1530 field emission SEM and a JEOL‐2100 TEM (JEOL GmbH, Germany) operated at 200 kV. XRD patterns were collected in Bragg‐Brentano geometry on a Bruker D8 Advance diffractometer with Cu Kα radiation using a zero‐background holder and a step size of 0.03° step^−1^ and a measuring time of 1 s step^−1^. N_2_ adsorption–desorption isotherms were conducted by using Quantachrome Autosorb‐1 systems at 77 K. Specific surface areas were calculated by using the Brunauer–Emmett–Teller (BET) method based on a multipoint analysis. The pore size distribution was estimated based on the Barrett, Joyner, and Halenda (BJH) method. XPS was conducted using a Thermo Scientific KAlpha spectrometer (monochromatic X‐ray source: Al Kα anode 1486.6 eV). TGA is carried out in a Netzsch TG 209F1 Iris under an Ar (or synthetic air) stream. The amount of the MoS_2_ in MoS_2_/CNFs was calculated using the formula wt% (MoS_2_) = wt% (MoO_3_) × M(MoS_2_)/M(MoO_3_). UV–vis absorption spectroscopy was recorded using a PerkinElmer Lambda 650 spectrometer at room temperature, with a DOL/DME (v/v = 1/1) mixture as the reference. The diameter distribution of MoS_2_/CNFs was analyzed using ImageJ software based on 50 individual nanofibers from an SEM image.

The as‐prepared MoS_2_/CNFs and CNFs were directly cut and used as the host materials. The diameter of the cathodes was 12.7 mm (the corresponding area was 1.27 cm^2^) and the average mass of the electrodes was 0.8–1.0 mg cm^−2^. The areal loading of the sulfur was controlled to be 1.0 mg cm^−2^ by adding 10 μL Li_2_S_8_ catholyte (0.5 m). 1.0 m LiTFSI in DOL/DME solution (V_DOL_: V_DME_ = 1:1) with 2.0 wt% of LiNO_3_ was used as the electrolyte. CR2025 coin cells were assembled with the Li foil as the anode and a piece of Celgard membrane as the separator in an Ar‐filled glove box (UNIlab plus, M. BRAUN) with H_2_O content < 0.5 ppm and O_2_ content < 0.5 ppm. The cathode and the anode sides were supplemented with 15.0 μL of electrolytes, respectively. The electrolyte‐to‐sulfur ratio (E/S) was 32 μL mg^−1^. Before the electrochemical testing, all the cells were aged at room temperature under open circuit potential for 12.0 h to let the electrolytes wet the electrodes. In this work, the current density of 1.0C equals 1675.0 mA g^−1^. The specific capacity is calculated based on the mass of sulfur. The galvanostatic charge and discharge were conducted on a Neware battery testing system at room temperature. The CV curves of the assembled coin cells were measured with a Biologic VMP3 electrochemical workstation.

## Conflict of Interest

The authors declare no conflict of interest.

## Supporting information

Supplementary Material

## Data Availability

The data that support the findings of this study are available from the corresponding author upon reasonable request.
