# Supplementary material for: Lignin Derived Free‐Standing Sulfur Host Functionalized with MoS2 for Li–S Batteries
Source: ChemSusChem. 2025 Oct 3;18(24):e202501698. doi: 10.1002/cssc.202501698 (PMC12703437; doi:10.1002/cssc.202501698)
Supplement: Supplementary file 1 — Supplementary Material [file CSSC-18-e202501698-s001.pdf]

Support Information for

Lignin Derived Free-Standing Sulfur Host Functionalized with MoS_2 for Li−S Batteries

Ping Feng[a,b], *Qingping Wu*[a,d], *Yael Rodriguez Ayllon*[a], *Yongchao Chen*[b], *Marius Hermesdorf*[b], *Martin Oschatz*[b,c], *Yan Lu*[a,b,c*]

P. Feng, Q. Wu, Y. Ayllon, Y. Lu

[a]Institute of Electrochemical Energy Storage, Helmholtz-Zentrum Berlin für Materialien und Energie, Berlin,14109, Germany.

P. Feng, Y. Lu, Y. Chen, M. Hermesdorf, M. Oschatz,

[b]Institute for Technical Chemistry and Environmental Chemistry, Friedrich-Schiller-Universität Jena, Jena, 07743, Germany.

M. Oschatz, Y. Lu

[c]Helmholtz Institute for Polymers in Energy Applications Jena (HIPOLE Jena), Jena, 07743, Germany.

Q. Wu

[d]Chongqing Institute of Green and Intelligent Technology, Chinese Academy of Sciences, Chongqing, 400714, China.

[*] Corresponding author. E-mail address: yan.lu@helmholtz-berlin.de (Yan Lu).

Experimental Section

Materials

Lignin was purchased from Suzano Papel e Cellulose S.A. in Brazil. Polyvinyl pyrrolidone (PVP, average Mw = 1,300,000), ammonium tetrathiomolybdate ($(NH_4)_2MoS_4$), N,N-Dimethylformamide (DMF, 99.8%), bis(trifluoromethane)sulfonimide lithium salt (LiTFSI, 99.95 %), 1,3-dioxolane (DOL, 99.0 %), 1,2-dimethoxyethane (DME, 99.0 %), lithium nitrate ($LiNO_3$, 99.99%), sublimed sulfur powder, lithium sulfide (Li_2S, 99.98%), polyvinylidene fluoride (PVDF) were purchased from Sigma-Aldrich. Lithium chips (diameter:14 mm, thickness: ~300 μm) were purchased from China Energy Lithium. Conductive carbon was purchased from MTI Corporation. All chemicals were used without any further purification.

Synthesis of MoS_2/CNFs

The MoS_2/CNFs were prepared by using the electrospinning method (Spinbox System, Bioinicia S.L.). The electrospinning solution was prepared by adding 3.8 g Lignin, 0.2 g polyvinyl pyrrolidone (PVP, average Mw = 1300000), and 0.5 g $(NH_4)_2MoS_4$ into 10 mL dimethylformamide and stirring for 12 h. This solution was loaded into a syringe with a needle and connected to a pump to control the flow rate. The distance between the needle and collector was 15 cm, and the electrospinning process was conducted at a constant flow rate of 2 ml/h under a voltage of 12 kV. Subsequently, the collected polymer nanofibers were peroxidized at 250 °C for 2 h in air. Then, the sample was heated to 800 °C with a ramp of 5 °C min^{-1} under a flow Ar atmosphere and maintained for 2 h to prepare the MoS_2/CNFs. The pure CNFs were prepared using the same method as MoS_2/CNFs, but without adding $(NH_4)_2MoS_4$.

Adsorption tests of LiPSs

The Li_2S_6 solution (0.2 M) was first prepared by the chemical reaction between sulfur and lithium sulfide ($Li_2S + 5S \rightarrow Li_2S_6$). In a typical process, 0.64 g sulfur and 0.18 g Li_2S (99.98%) were dissolved in 20.0 mL DOL/DME solution (V_{DOL}: V_{DME} = 1: 1) in a 50.0 mL bottle and kept stirring overnight in an Ar-filled glove box. Then this suspension was heated at 80 °C in a vacuum oven inside the glove box for one day to yield the Li_2S_6 solution (0.2 M) with red-brown color. This 0.2 M Li_2S_6 solution was diluted to 2.0 mM for further use. After

that, the MoS_2/CNFs and CNFs with the same mass (20.0 mg) were separately added to 2.0 mM Li_2S_6 solution (4.0 mL). After aging for 3 hours inside the glove box, the supernatant liquid was sealed in cylinder quartz for the UV-vis absorption spectroscopy test. The MoS_2/CNFs after Li_2S_6 adsorption tests were washed with DOL solution and dried in the glove box for further XPS measurements.

Kinetics of Li_2S precipitation on the host materials

The Li_2S_8 catholyte was prepared by the chemical reaction between sulfur and lithium sulfide ($Li_2S + 7S \rightarrow Li_2S_8$). In a typical process, 4.48 g sulfur and 0.92 g Li_2S (99.98%) were dissolved in 20.0 mL DOL/DME solution (V_{DOL}: V_{DME} = 1: 1) with 114.8 mg $LiNO_3$ additives in a 50.0 mL bottle and kept stirring overnight in an Ar-filled glove box. Then this suspension was heated at 80 °C in a vacuum oven inside the glove box for one day to yield the Li_2S_8 catholyte (0.5 M) with red-brown color. For the Li_2S precipitation test, the electrode was prepared by casting the slurry of MoS_2/CNFs (or CNFs), conductive carbon, and PVDF (7:2:1 in weight ratio) on carbon paper by the doctor blade technique. After drying at 50 °C under vacuum overnight, the electrode was cut into wafers with a diameter of 12.7 mm. The coin cell was assembled with the MoS_2/CNFs (or CNFs) as the cathode, Li as the anode, and a Celgard membrane serving as the separator. The loading of Li_2S_8 catholyte for the Li_2S precipitation test is 1 mg cm^{-2}. The cathode and the anode sides were supplemented with 15.0 μL electrolytes, respectively. All the assembled coin cells were aged at room temperature for 12.0 h. After that, the cell was first discharged galvanostatically at 0.1 C to 2.12 V, then discharged potentiostatically at 2.05 V for Li_2S nucleation and growth. The current *vs.* time curve was collected for kinetic analysis. For the symmetrical cell, two identical electrodes (MoS_2/CNFs or CNFs) were assembled into a CR2032 coin cell. 1.0 M Li_2S_8 catholyte (5.0 μL) was loaded to the host electrodes as the sulfur source. The cathode and the anode sides were supplemented with 15.0 μL electrolytes, respectively. CV measurements of the symmetric cell were performed at a scan rate of 10.0 mV s^{-1} within the potential range from −1.0 to 1.0 V.

Electrochemical measurements

The as-prepared MoS_2/CNFs and CNFs were directly cut and used as the host materials. The diameter of the cathodes was 12.7 mm (the corresponding area was 1.27 cm^2) and the

average mass of the electrodes was 0.8–1.0 mg cm^{-2}. The areal loading of the sulfur was controlled to be 1.0 mg cm^{-2} by adding 10 μL Li_2S_8 catholyte (0.5 M). 1.0 M LiTFSI in DOL/DME solution (V_{DOL} : V_{DME} = 1:1) with 2.0 wt. % of $LiNO_3$ was used as the electrolyte. CR2025 coin cells were assembled with the Li foil as the anode and a piece of Celgard membrane as the separator in an Ar-filled glove box (UNIlab plus, M. BRAUN) with H_2O content < 0.5 ppm and O_2 content < 0.5 ppm. The cathode and the anode sides were supplemented with 15.0 μL electrolytes, respectively. The electrolyte-to-sulfur ratio (E/S) was 32 μL mg^{-1}. Before the electrochemical testing, all the cells were aged at room temperature under open circuit potential for 12.0 h to let the electrolytes wet the electrodes. In this work, the current density of 1.0 C equals 1675.0 mA g^{-1}. The specific capacity is calculated based on the mass of sulfur. The galvanostatic charge and discharge were conducted on a Neware battery testing system at room temperature. The CV curves of the assembled coin cells were measured with a Biologic VMP3 electrochemical workstation.

Characterization

The morphology of the obtained samples was investigated by a LEO 1530 field emission SEM and a JEOL-2100 TEM (JEOL GmbH, Germany) operated at 200 kV. X-ray diffraction (XRD) patterns were collected in Bragg-Brentano geometry on a Bruker D8 Advance diffractometer with Cu Kα radiation using a zero-background holder and a step size of 0.03 °/step and a measuring time of 1 s/step. N_2 adsorption-desorption isotherms were conducted by using Quantachrome Autosorb-1 systems at 77 K. Specific surface areas were calculated by using the Brunauer-Emmett-Teller (BET) method based on a multipoint analysis. The pore size distribution was estimated based on the Barrett, Joyner, and Halenda (BJH) method. X-ray photoelectron spectroscopy (XPS) was conducted using a Thermo Scientific KAlpha spectrometer (monochromatic X-ray source: Al Kα anode 1486.6 eV). Thermogravimetric analysis (TGA) is carried out in a Netzsch TG 209F1 iris under an Ar (or synthetic air) stream. The amount of the MoS_2 in MoS_2/CNFs was calculated using the formula wt% (MoS_2) = wt% (MoO_3) × M(MoS_2)/M(MoO_3). UV-vis absorption spectroscopy was recorded using a PerkinElmer Lambda 650 spectrometer at room temperature, with a DOL/DME (v/v = 1/1) mixture as the reference. The diameter distribution of MoS_2/CNFs was

analyzed using ImageJ software based on 50 individual nanofibers from SEM image.

Figures

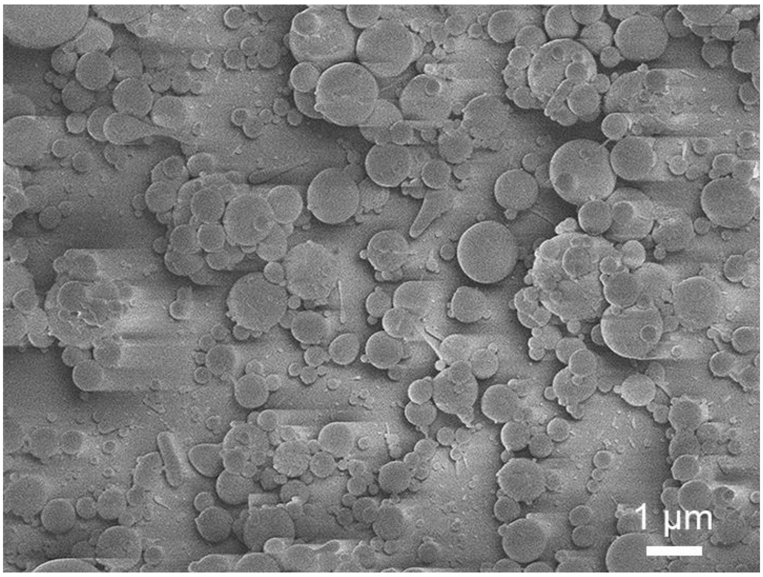

Figure S1. SEM images of the lignin nanospheres obtained by electrospinning of 20 wt.% pure lignin solution.

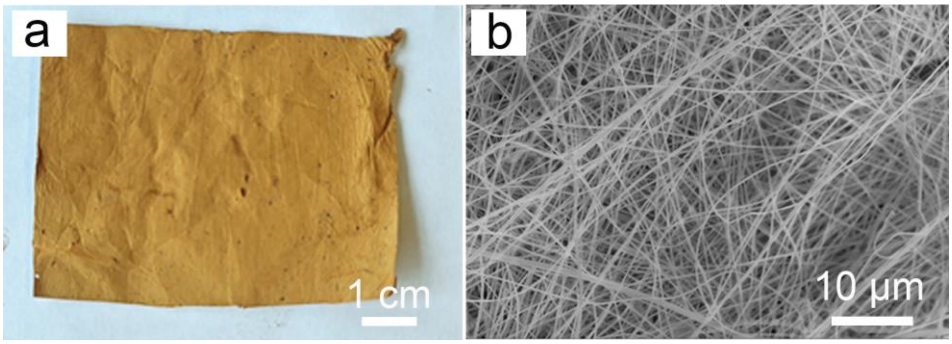

Figure S2. (a) Digital and (b) SEM images of the $(NH_4)_2MoS_4$@lignin/PVP nanofibers.

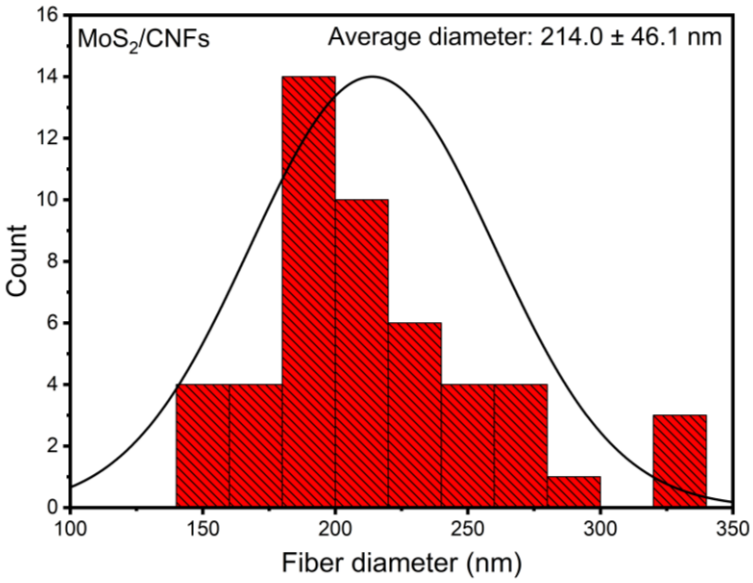

Figure S3. Statistical analysis of the diameter for the MoS_2/CNFs based on analysis of 50 individual nanofibers from the SEM images using ImageJ software.

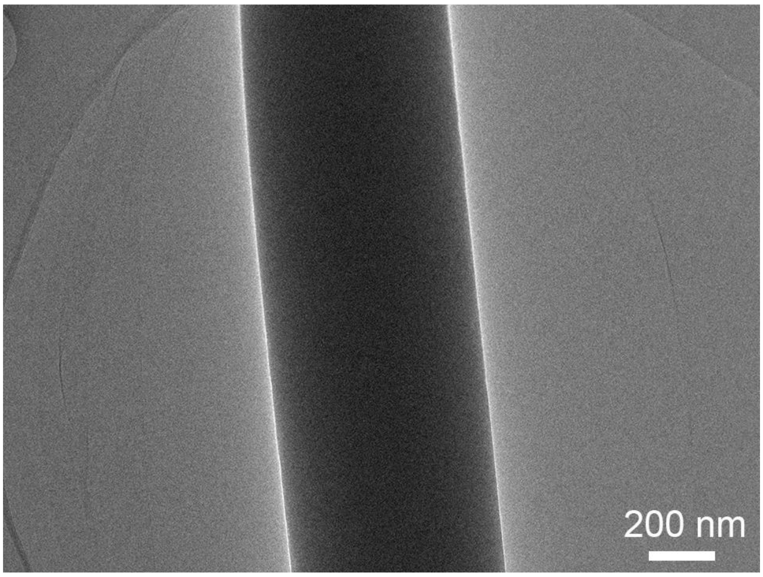

Figure S4. TEM image of the pure CNFs.

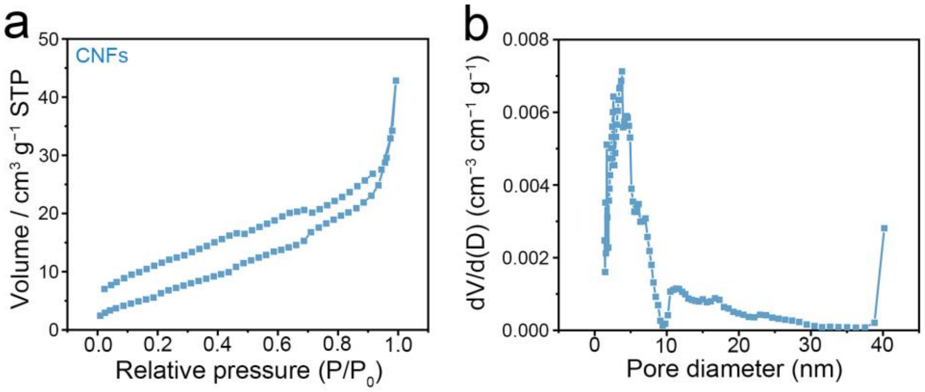

Figure S5. (a) Nitrogen adsorption−desorption isotherms of the CNFs. (b) The corresponding pore size distribution plot of the CNFs.

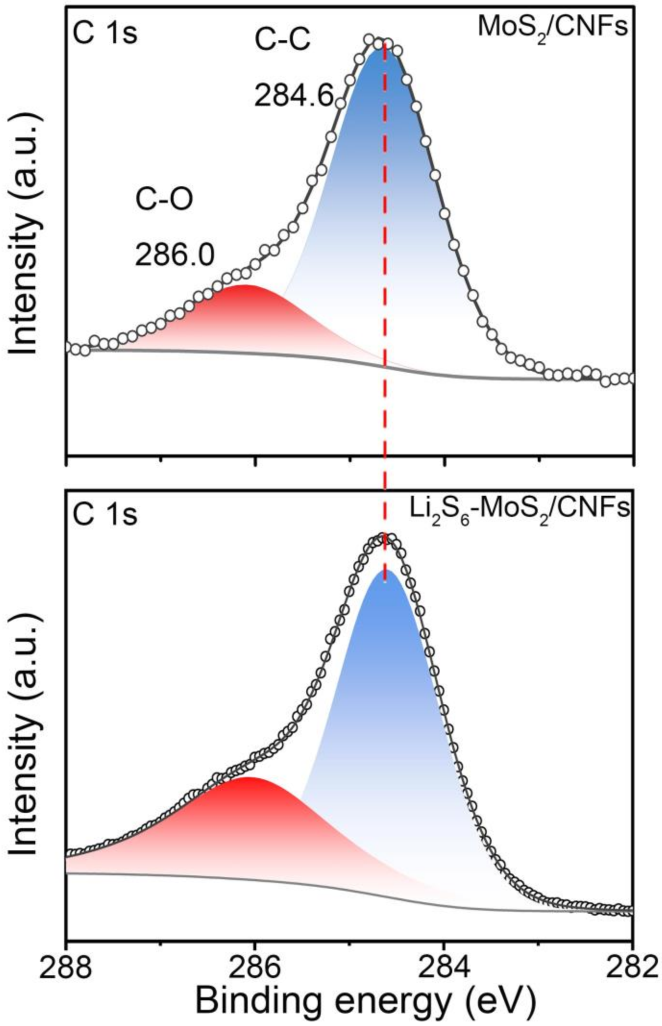

Figure S6. C 1s XPS spectra of MoS_2/CNFs before (top) and after (below) Li_2S_6 adsorption test.

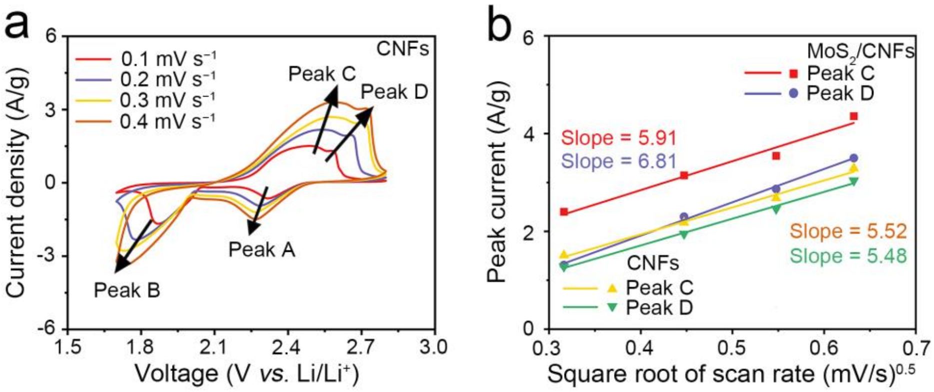

Figure S7. (a) CV curves of the coin cell with the CNFs-based electrodes in the voltage range of 1.7−2.8 V at various scan rates of 0.1, 0.2, 0.3, and 0.4 mV s^{-1}. (b) Plot of CV peak current of the peak C ($Li_2S \rightarrow Li_2S_4$) and peak D ($Li_2S_4 \rightarrow S_8$) versus the square root of scan rate.

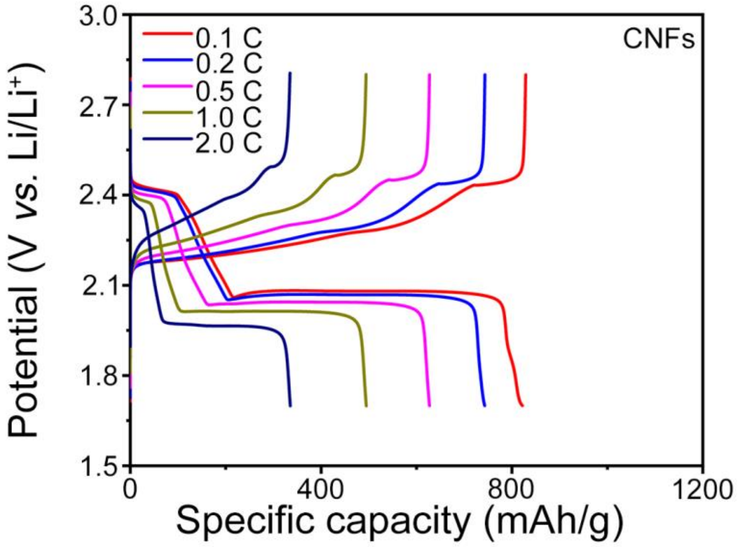

Figure S8. Charge/discharge curve profiles of CNFs-based Li−S cells at various rates from 0.1 to 2 C.

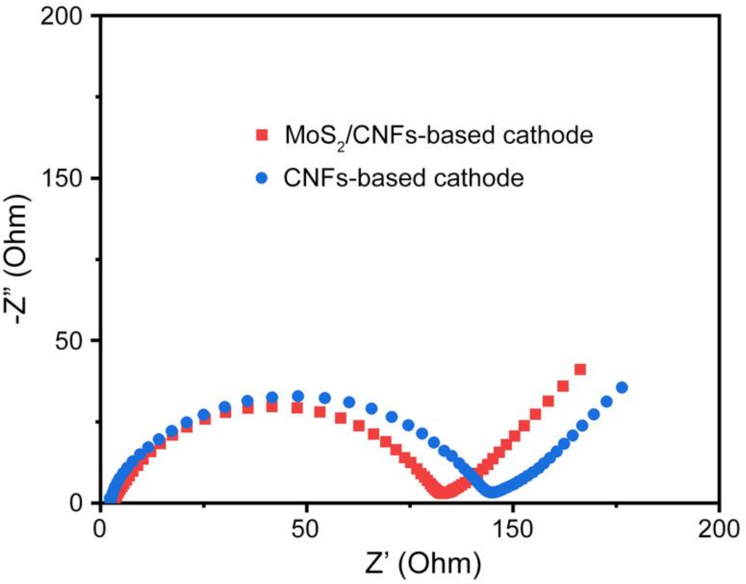

Figure S9. Electrochemical impedance spectroscopy of Li−S batteries with MoS_2/CNFs and CNFs-based electrodes.

Table S1. Comparison of peak voltages and peak currents from the CV results of MoS_2/CNFs and CNFs-based electrodes in Fig. 4b.

Peak position(V)	A	B	C	D
MoS_2@CNFs	2.35	1.95	2.42	2.49
CNFs	2.32	1.87	2.49	2.56
Current densities (A/g)	A	B	C	D
MoS_2@CNFs	-0.72	-2.15	2.42	1.31
CNFs	-0.65	-1.67	1.52	1.31

Table S2. Comparison of the electrochemical performance of different cathodes in Li−S batteries.

Carbon source	Host materials	Self-supporting	Sulfur loading (mg cm^{-2})	Electrolyte to sulfur ratio (μL mg^{-1})	Current density (C)	Initial capacity (mAh g^{-1})	Final capacity (mAh g^{-1})	Cycle number	Ref.
Lignin	MoS_2/CNFs	Yes	1.0	32	1.0	715.3	609.3	200	This work
Lignin	CNT film	Yes	1.0	20	0.5	665.4	435.3	200	[S1]
Lignin	NS@LDPC	No	2.8	-	0.1	1100.0	509.3	110	[S2]
Lignin	LPC/CNTs	No	1.0	-	1.0	673.4	582.8	400	[S3]
Potato starch	rGO@HYC	No	1.4−2.0	20−28	0.5	617	463	500	[S4]
Fluffy catkins	TACM	No	1.5	20	0.5	697	540	500	[S5]
Sugar cane	NDPCs	No	1.0	20	0.5	926	571	400	[S6]
Carbon cloth	MoO_2/MoS_2@CC	Yes	4	10-15	1	909	640	140	[S7]
Polypyrrole	CNT@HCNF@MoS_2	Yes	2	12	2	650	733	250	[S8]

Reference

[S1] F. Liu, P. Feng, M. Yuan, G. Zhai, M. T. Innocent, H. Xiang, Q. Wu, Y. Lu, M. Zhu, *ACS Sustain. Chem. Eng.* **2023**, *11*, 16544-16553.

[S2] Y. Tian, Z. Yang, H. Wang, W. Xiong, X. Lin, S. Wang, F. Kong, P. Li, Y. Xi, F. Zhang, Q. Li, *J. Power Sources* **2024**, *621*, 235322.

[S3] S. Zeng, L. Lin, Y. Li, J. Peng, Y. Wen, Z. Liang, Z. He, L. Lan, Y. Huang, W. Xu, *J. Mater. Sci.* **2025**, *60*, 2960-2971.

[S4] Y. Li, Y. Cai, Z. Cai, J. Xu, J. Sonamuthu, G. Zhu, J. Militky, W. Jin, J. Yao, *Electrochim. Acta* **2018**, *285*, 317-325.

[S5] L. Fan, Z. Li, W. Kang, B. Cheng, *Renew. Energ.* **2020**, *155*, 309-316.

[S6] S. Wang, K. Zou, Y. Qian, Y. Deng, L. Zhang, G. Chen, *Carbon* **2019**, *144*, 745-755.

[S7] Y. Tang, Y. Huang, L. Luo, D. Fan, Y. Lu, A. Manthiram, *Electrochim. Acta* **2021**, *367*, 137482.

[S8] Z. Zhao, W. Feng, W. Hu, Y. Niu, W. Su, X. Zheng, L. Zhang, *J. Electroanal. Chem.* **2024**, *971*, 118559.
